# Simultaneous wood and metal particle detection on dark-field radiography

**DOI:** 10.1186/s41747-017-0034-1

**Published:** 2018-01-10

**Authors:** Eva-Maria Braig, Lorenz Birnbacher, Florian Schaff, Lukas Gromann, Alexander Fingerle, Julia Herzen, Ernst Rummeny, Peter Noël, Franz Pfeiffer, Daniela Muenzel

**Affiliations:** 1 Chair of Biomedical Physics, Department of Physics and Munich School of BioEngineering, Technical University of Munich, James-Franck-Str. 1, 85748 Garching, Germany; 2 Department of Diagnostic and Interventional Radiology, Klinikum rechts der Isar, Technical University of Munich, Ismaninger Straße 22, 81675 München, Germany; 3 Institute for Advanced Study, Technical University of Munich, 85748 Garching, Germany

**Keywords:** Radiography, Foreign bodies, Extremities, Hand, Dark-field contrast

## Abstract

**Background:**

Currently, the detection of retained wood is a frequent but challenging task in emergency care. The purpose of this study is to demonstrate improved foreign-body detection with the novel approach of preclinical X-ray dark-field radiography.

**Methods:**

At a preclinical dark-field x-ray radiography, setup resolution and sensitivity for simultaneous detection of wooden and metallic particles have been evaluated in a phantom study. A clinical setting has been simulated with a formalin fixated human hand where different typical foreign-body materials have been inserted. Signal-to-noise ratios (SNR) have been determined for all test objects.

**Results:**

On the phantom, the SNR value for wood in the dark-field channel was strongly improved by a factor 6 compared to conventional radiography and even compared to the SNR of an aluminium structure of the same size in conventional radiography. Splinters of wood < 300 μm in diameter were clearly detected on the dark-field radiography. Dark-field radiography of the formalin-fixated human hand showed a clear signal for wooden particles that could not be identified on conventional radiography.

**Conclusions:**

x-ray dark-field radiography enables the simultaneous detection of wooden and metallic particles in the extremities. It has the potential to improve and simplify the current state-of-the-art foreign-body detection.

## Key points

- Retained wood is hardly detectable in an effective, reliable way for emergency settings.
- Grating-based x-ray dark-field imaging enables the detection of wood on plain radiography.
- The signal-to-noise ratio of wood was six times higher compared to that obtained for conventional attenuation.
- Conventional radiography image is acquired simultaneously with the dark-field image.

## Background

Open wound care is one of the most frequent tasks in emergency care, while being closely related to the possibility of retained foreign bodies [1, 2]. Typically, for a patient with characteristic signs of a retained foreign body, physical examination is followed by a routine radiography [3].

The majority of foreign bodies consist either of metal, glass or wood [4, 5]. In particular, wood, the most frequent type of foreign body in the extremities, is likely to be missed in 85–95% of patients at the initial radiographic examination because of its low attenuation property [1, 3, 4, 6–9]. While x-ray radiography is highly sensitive for the detection of materials with high atomic numbers like metals, weakly absorbing foreign bodies are reportedly elusive in plain radiography. Even computed tomography (CT) does often not allow to distinguish wood from other hypodense materials like fat or air [6, 7, 9, 10].

Magnetic resonance imaging exhibits good soft-tissue contrast mainly due to differences in water and lipid contents as well as in relaxation times. However, routine foreign-body detection with magnetic resonance imaging is not feasible in an emergency department workflow due to poor accessibility, low cost-effectiveness, and particularly the risk for patients with possibly ferromagnetic foreign bodies associated with the strong magnetic field [11, 12].

Ultrasonography shows good success in detecting retained soft-tissue materials in the cases with a sufficient change of material impedance but is still highly dependent on the operator’s expertise and requires a time-consuming examination by the treating physician [3, 13].

In this proof-of-principle study, we present the simultaneous detection of metallic and wooden foreign bodies on preclinical x-ray dark-field radiography. The underlying technique is based on the detection of small angle scattering at micron-range substructures while simultaneously providing the conventional attenuation image [14–16]. In the following, we demonstrate experimentally the potential of foreign-body detection in x-ray dark-field radiography. Thereby, we show phantom and sample measurements with a preclinical setup and discuss the potential of diagnostic improvement of x-ray dark-field radiography for the detection of foreign bodies.

## Methods

### Signal extraction in x-ray dark-field imaging

**Fig. 1:**
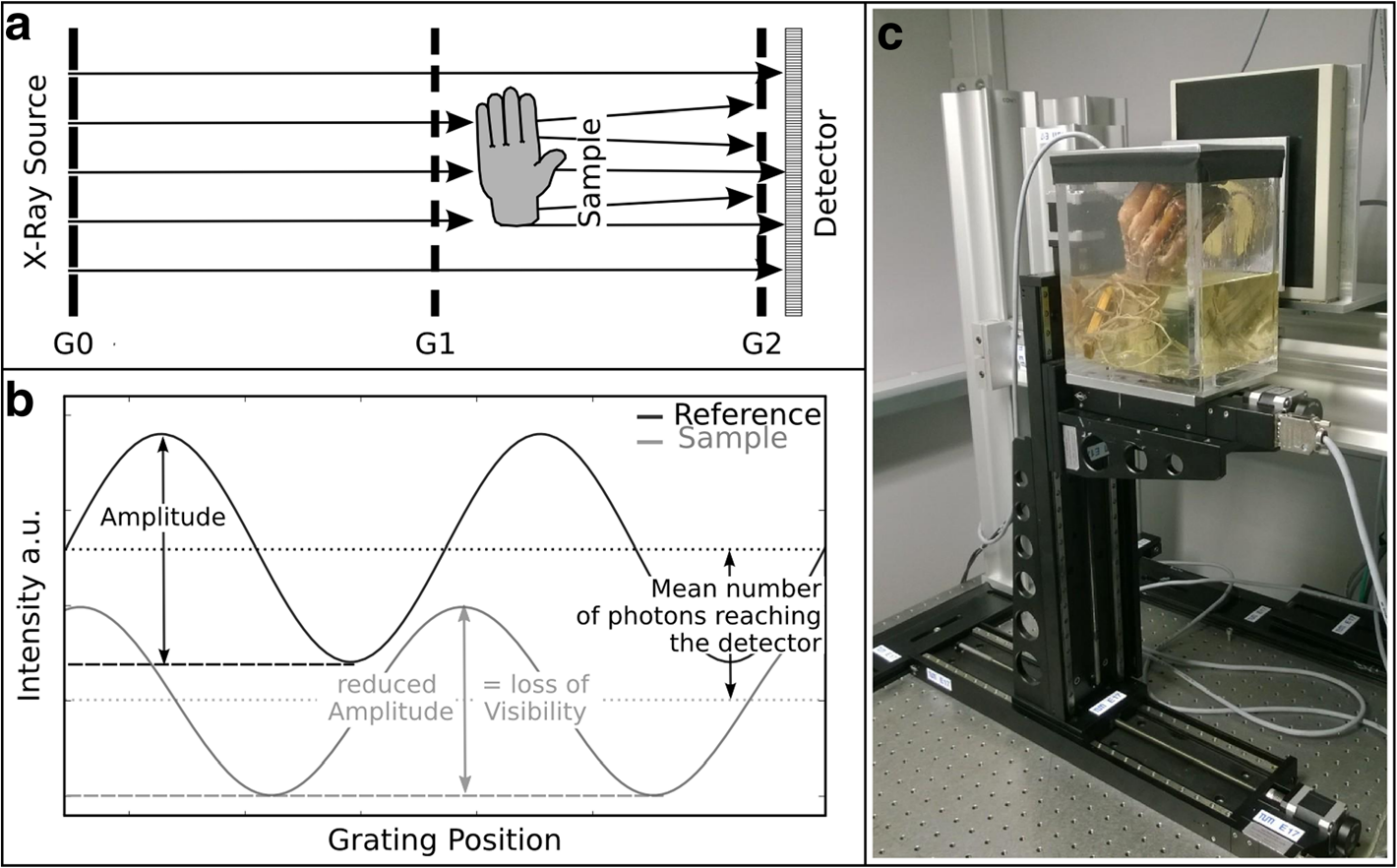
Basic principle of the experimental x-ray dark-field radiography setup. **a** The experimental dark-field radiography setup is based on a three grating interferometer, a so-called Talbot-Lau interferometer. The source grating G0 provides the necessary beam coherence for interference effects to occur with a clinical x-ray source, the phase grating G1 causes a periodic modulation of the interference pattern, and the analyser grating G2 enables the resolution of the pattern with a conventional detector. **b** The phase grating is shifted laterally in seven discrete steps leading to a resulting stepping curve for each detector pixel. Performing two scans, one without the object (reference scan) and one with the object (sample scan), leads to two different stepping curves. The conventional attenuation image is represented by the relative mean values of the curve and the relative reduction of the amplitude (which represents the visibility of the system) is a measure for the dark-field signal. **c** A human hand fixed in formalin was measured in preclinical x-ray dark-field setup to evaluate the diagnostic value for foreign-body detection

The x-ray dark-field radiography setup is based on a Talbot-Lau interferometer [16] consisting of an x-ray source, three x-ray gratings and one x-ray detector, as illustrated in Fig. 1. A source grating (G0) renders the method accessible with a clinical x-ray source by providing the beam coherence necessary for the method. A phase shifting grating (G1) causes a specific periodic interference pattern, which can be resolved by the analyser grating (G2) in combination with the detector. By laterally scanning one of the gratings (the so-called phase-stepping), one measures an oscillating signal for each single detector pixel. Comparing a sample scan to a reference scan without sample yields three complementary signals for the sample simultaneously: the conventional attenuation; the differential phase shift; and the dark-field signal. In this study, we focus mainly on the attenuation and the dark-field signal. A more detailed description of this method was previously given [14, 17–19].

### Technical parameters

The preclinical x-ray dark-field setup used a tungsten target x-ray tube (x-ray WorX SE 160, Garbsen, Germany), operating at 60 kVp and a power of 150 W. The detector was a Varian Paxscan 2520DX flatpanel detector with a CsI scintillator and a pixel size of 127 μm (Varian medical systems, Palo Alto, CA, USA). Due to cone-beam magnification, the effective pixel size was 110 μm. The interferometer was designed for a mean energy of 45 keV with grating periods of 10.0/5.0/10.0 μm for G0/G1/G2, respectively. The full field-of-view needed for the radiography of the hand was stitched together from 3 × 3 single images as the currently available size of the gratings is limited. The radiation exposure for the hand was 87.5 mAs distributed over seven grating steps for 5 s of exposure per step. The exposure time was varied between 1, 2, and 5 s. The incident air kerma at the position of the sample was measured with a PTW NOMEX dosimeter (PTW Freiburg GmbH, Germany). Considering all gratings and the setup geometry, a measured value of 0.22 mGy corresponds to an estimated image receptor dose of about 25 μGy for 1-s exposure time.

### Foreign-body phantom

**Fig. 2:**
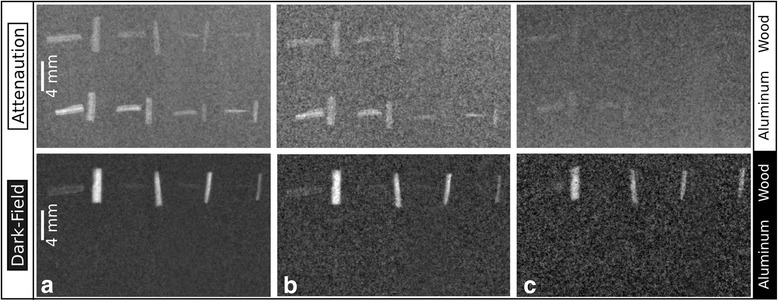
Phantom study for foreign-body detection with x-ray dark-field radiography. The test structures made of aluminium and wood were prepared onto a PMMA plate with 1.8 cm thickness for phantom configuration 1. The diameters of the structures were about 0.5, 0.7, 0.9, and 1.1 mm increasing from right to left. We aligned two splinters of each size perpendicularly to each other. In all images, the wooden test structures are on the top and the aluminium structures are on the bottom. The conventional attenuation is displayed on the upper row, the x-ray dark-field images on the lower row. **a** The radiographies at 5s exposure time per step. The same configuration has been imaged with 1s exposure time (**b**). **c** The phantom configuration 2 (i.e. with an additional aluminium plate) at 2s exposure time per step

The phantom was specifically designed for testing the sensitivity and resolution of the method for very small particles. It consisted of an 18 mm thick polymethyl methacrylate (PMMA) plate and pairwise test objects of aluminium and wood with diameters of 0.5, 0.7, 0.9, and 1.1 mm fixed on the PMMA plate with tape (configuration 1, see Fig. 2), pairwise horizontally and vertically aligned relative to the grating orientation. The PMMA plate was chosen to mimic the attenuation characteristics of soft tissue.

In the second step, the configuration of the above described phantom was extended by an aluminium-oxide plate of 1 cm (configuration 2), which mimics the attenuation of bone [20–22]. Images were acquired in both phantom configurations to verify the signal intensity in the intercarpal space as well as directly behind a bone.

Two images were acquired for configuration 1 with exposure times of 1 and 5 s per step. The exposure time for configuration 2 was 2 s per step.

### Specimen preparation

The study was approved by the local institutional review board. The donor of the hand used in this study had given his body for educational and research purposes and provided written informed consent before death, in compliance with local institutional and legal requirements. The hand was fixed with formaldehyde solution before the experiments.

We inserted different typical wooden and metallic foreign objects in the human hand (Fig. 1c). For the measurement, the specimen was placed into a planar plastic container and the formalin level was reduced. The human hand was imaged with an exposure time of 5 s per step.

### Visual foreign-body detection

All images were acquired using the above described preclinical setup with the same technical parameters. No specific post-processing has been applied. The stitched radiographic images were analysed by two trained radiologists (DM, AF), both of them with more than ten years of experience.

### Signal-to-noise analysis

The signal-to-noise ratio (SNR) was calculated (NumPy package, Python 2.7) for all foreign-body objects in the specimen and the phantom for a region of interest $\overline{\mathrm{s}}$ inside the object compared to the mean of a region of interest in the background $\overline{\mathrm{b}}$ and the standard deviation of this background region σ_b_ according to the following formula:

$$ SNR=\frac{\left|\overline{\mathrm{s}}-\overline{\mathrm{b}}\right|}{\sigma_{\mathrm{b}}}. $$

An SNR value < 1 means that an object cannot be distinguished from the background. Thus, all values < 1 were set to zero.

## Results

The test features will be referred to by numbers *1–4* with feature *1v/h* the smallest (0.5 mm) vertical/horizontal feature and feature *4v/h* the largest (1.1 mm) vertical/horizontal feature. The radiographic attenuation and dark-field images for different exposure times and two different phantom configurations are presented in Fig. 2.

### Visual evaluation of dark-field radiography images of the phantom

In the attenuation-based image there was no difference in signal strength between visible vertical and horizontal particles. At 5s exposure time (Fig. 2a), all wooden and aluminium test objects could be detected on the flat background. For 1s exposure time (Fig. 2b), only the larger wooden particles *3* and *4* and all aluminium features except feature *2v* were detectable. Behind the bone-like absorber (Fig. 2c), only the largest horizontal wood and aluminium particles *4 h* were slightly visible.

In the dark-field image there was a clear difference between vertical and horizontal particles. At 5s exposure time, all vertical wooden objects were clearly detectable. The horizontal features 2 h, 3 h, 4 h were slightly visible. At 1s exposure time, still all vertical wooden particles and the largest horizontal wooden particle could be identified. Behind the bone-like absorber, all vertical wooden objects could be identified. On the dark-field images, none of the aluminium particles was detected.

Both radiologists provided the same results for the independent visual inspection for all particles.

### SNR analysis in phantom measurements

Results of the SNR analysis for all test objects are displayed in Table 1, where SNR values have been calculated from the image data shown in Fig. 2.

**Table 1:** SNR values for all horizontal (h) and vertical (v) test objects in the phantom for the different phantom configurations 1 (without the additional bone-like absorber) and 2 (with the additional bone-like absorber) at different exposure times

SNR values for wooden and metallic objects in attenuation and dark-field						
Attenuation						
Phantom	Config 1		Config 2	Config 1		Config 2
Exposure (s)	5	1	2	5	1	2
Test objects	Wood			Aluminium		
1 h	1.7	1.1	0	4.9	2.7	0
1 v	1.7	0	0	4.3	2.1	0
2 h	2.6	0	0	3.2	1.5	0
2 v	2.5	1.3	0	1.9	0	0
3 h	2.5	1.6	0	6.5	3.1	1.4
3 v	3.1	1.0	0	4.6	2.4	1.4
4 h	4.9	2.0	0	8.2	3.7	1.4
4 v	4.6	2.2	0	8.4	3.9	1.9
Dark-field						
Phantom	Config 1		Config 2	Config 1		Config 2
Exposure (s)	5	1	2	5	1	2
Test objects	Wood			Aluminium		
1 h	1.6	0	0	0	0	0
1 v	7.5	3.1	3.7	0	0	0
2 h	1.9	0	0	0	0	0
2 v	19.4	8.6	3.4	0	0	0
3 h	1.6	0	0	0	0	0
3v	18.7	8.7	3.8	0	0	0
4 h	3.3	1.6	0	0	0	0
4 v	25.2	11.4	5.4	0	0	0

In the attenuation-based image obtained with 5s exposure time per step, the wooden particles showed SNR values in the range of 1.7–4.9. For the aluminium objects, the SNR was in the range of 1.9–8.4. At 1-s exposure time, the SNR was in the range of 0–2.2 for the wooden objects and 0–3.9 for the aluminium particles. With an additional bone-like absorber in the phantom, the SNR decreased to 0 for all wooden features. Only the larger aluminium particles showed an SNR value slightly > 1, in the range of 1.4–1.9.

In the dark-field modality, the horizontal and vertical features showed a different behaviour in accordance with the directional sensitivity of the interferometer. At 5s exposure time, the vertical wooden features showed SNR values in the range of 7.5–25.2, the horizontal ones in the range of 1.6–3.3. At 1s exposure time, the SNR values for the vertical wooden objects were in the range of 3.1–11.4; only the largest horizontal wood particle had an SNR > 1 with a value of 1.6. In all dark-field images, the aluminium particles presented with an SNR value < 1 and were therefore set to zero. Behind the bone-like absorber, the SNR values for the vertical wooden particles were in the range of 3.4–5.4 and for the horizontal particles the SNR was 0 as well as for all aluminium particles.

Direct comparison of the SNR values of wooden particles on dark-field radiography and conventional radiography showed an average SNR increase of a factor of 6 and an increase from no signal to an average SNR of 4.1 for the features behind the bone-like absorber.

### Visual evaluation of the dark-field radiography of the human hand

**Fig. 3:**
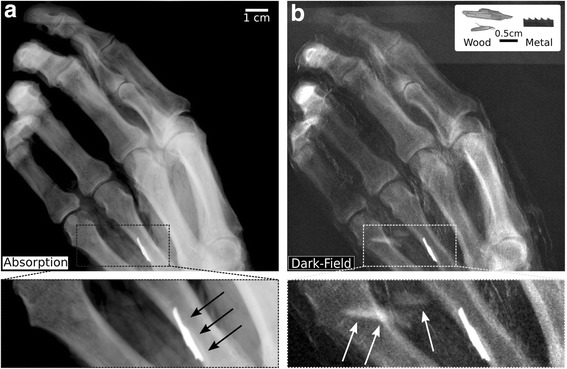
Foreign-body detection with x-ray dark-field radiography of a human hand. x-ray dark-field imaging allows for the simultaneous acquisition of a conventional attenuation image (**a**) and a dark-field image (**b**) of a human hand fixed in formalin. The images (antero-posterior projection) show the metacarpal and phalangeal bones of the right hand. Two wooden particles and one metallic particle were inserted into the hand to mimic foreign bodies, as shown in the inlay on the upper right. The metallic foreign body is lodged in the soft tissue of the palmar hand in front of the diaphysis of the IV metacarpal bone while the two wooden foreign bodies are located in front of the V metacarpal bones and the intermetacarpal space IV/V, respectively. High atomic number materials like the metallic saw blade provide a strong signal in the attenuation channel as indicated by the black arrows in the magnified attenuation image. However, the x-ray attenuation contrast of dry wood is poor and therefore the wooden test objects are difficult to detect in the attenuation image. In contrast, the dark-field signal is sensitive to small angle scattering at structures at a micron range occurring in wood. Both wooden objects can clearly be detected in the dark-field image which are marked with white arrows in the magnified view of the dark-field image

In Fig. 3, the dark-field radiography image of a formalin-fixed human hand is presented, which reveals simultaneously the conventional attenuation (Fig. 3a) and the dark-field signal (Fig. 3b). The phalangeal and metacarpal bones of the right hand are imaged with an anteroposterior projection. The metallic test object was a small piece of a saw blade. It can clearly be identified in the conventional attenuation image lateral of the IV metacarpal bone. The wooden test objects cannot be identified on the attenuation-based image.

In the dark-field channel the smaller wooden test particle can be detected in the IV interdigital space. Right next to it there is the larger wooden particle, partially lying behind the V metacarpal bone. Both wooden particles cause a strong signal and can be identified as foreign bodies. The dark-field signal of the wood is still strong behind the bone.

## Discussion

In this proof-of-principle study, we demonstrated, in a preclinical setting, the potential of x-ray dark-field radiography for an improved detection of wooden foreign bodies. We found that the dark-field signal for wooden particles is related to an even higher signal level than the corresponding signal of aluminium on conventional radiography. In dark-field radiography, the simultaneous image information of the attenuation and the small angle scattering allows for an improved assessment of foreign bodies.

The phantom study showed a high sensitivity of this method that allowed the visualisation of even very small structures in the size range of a few detector pixels, i.e. in the shown configuration, a few hundred microns. By mimicking the attenuation characteristics of bone and soft-tissue in the phantom with aluminium and PMMA, we evaluated the sensitivity of the dark-field signal in a realistic setting. As the dark-field contrast originates from small angle scattering of substructures at the micron range [18], it is less affected by the superimposition of an additional absorber in the direct beam path in contrast to the attenuation signal from, for example, metallic objects. Further, we emphasise that the different origin of the simultaneously acquired image signals guarantees their complementarity. Thus, this technique has the potential to characterise the quality of the retained bodies in a single x-ray exposure, which is likely to simplify the removal procedure of the foreign body and the planning of an optimal management [23, 24].

The strong directional dependency of the dark-field signal allows for a precise localisation of the objects even in projection. This helps for the preparation of surgical removal as well as the evaluation of the risk for relating anatomic structures as can be seen with the exemplary dark-field radiography of the human hand.

Current research in the field of biomedical x-ray dark-field imaging shows many promising applications of the technique, although still in a preclinical phase [25–29].

For the presented case of foreign-body detection, the isolated distal position of hands and feet reduces the requirements for a dark-field scanner in terms of x-ray energy, grating specifications and exposure time. The bones of the hand and fingers are also visible in the dark-field images. In our study, with a normal anatomical structure of the bones, we did not obtain any additional information from dark-field images. Thüring et al. [30] presented an overview on the presentation of the bony structure of the hand in dark-field imaging. Further information on the anatomical structure of the bones can be assessed with grating-based x-ray vector radiography [31].

As the presented setup is in a preclinical stage and not optimised for clinical application, we focused on demonstrating a proof-of-principle application of x-ray dark-field radiography for foreign-body detection. In particular, the limited field of view and the comparably low photon flux at the microfocus tube, coming with a high acquisition time in our stitching approach, leave room for improvements. Optimisation of the x-ray spectrum and the acquisition approach as well as an increased field of view will be the next steps to reduce the x-ray dose and the measurement time for clinical applications.

Of note, no dedicated post-processing has been applied and the shown images are, besides the stitching, raw images. Compared to the applicable guidelines for radiography of the hand, the image receptor dose exceeds the suggested value by a factor of 2.5 for 1-s exposure time per step [32]. A larger pixel size and the use of a state-of-the-art clinical radiography detector would allow for a significant dose reduction. As the used setup was only available with the described components, we designed the phantom to demonstrate the strong dark-field signal of wood in direct comparison to the attenuation signal of aluminium. At a photon-statistics where the smallest aluminium particle in the conventional image disappears on the background noise, a wooden splinter of the same size is still clearly visible on the dark-field image.

Together with the ongoing progress in technical improvement of the x-ray dark-field method and its adaptation for clinical applications we think that these results obtained using an experimental x-ray dark-field scanner radiography setup, illustrate the clinical potential of the method for emergency wound care due to improved and complementary foreign-body detection. Considering the high frequency and the minor emergency character of foreign-body detection in human hands and feet, the method can close a gap between insufficient visual inspections and elaborate three-dimensional imaging methods like computed tomography and magnetic resonance imaging.

In conclusion, we demonstrated the high potential of dark-field radiography for the detection of wooden foreign bodies in a phantom and a human hand specimen. Thus, we assume that this method will have the potential to simplify the diagnostic workflow and to increase the success of foreign-body detection.

## Abbreviations

PMMA: Polymethyl methacrylate
SNR: Signal-to-noise ratio

## References

[1] Ebrahimi A, Radmanesh M, Rabiei S (2013). Surgical removal of neglected soft tissue foreign bodies by needle-guided technique. Iran J Otorhinolaryngol.

[2] Davis J, Czerniski B, Au A (2015). Diagnostic accuracy of ultrasonography in retained soft tissue foreign bodies: A systematic review and meta-analysis. Acad Emerg Med.

[3] Turkcuer I, Atilla R, Topacoglu H (2006). Do we really need plain and soft-tissue radiographies to detect radiolucent foreign bodies in the ED?. Am J Emerg Med.

[4] Levine MR, Gorman SM, Young CF (2008). Clinical characteristics and management of wound foreign bodies in the ED. Am J Emerg Med.

[5] Lewis D, Jivraj A, Atkinson P (2015). My patient is injured: identifying foreign bodies with ultrasound. Ultrasound.

[6] Becton JL, Christian JDJ (1977). Foreign bodies in the hand. South Med J.

[7] Anderson MA, Newmeyer WL, Kilgore ES (1982). Diagnosis and treatment of retained foreign bodies in the hand. Am J Surg.

[8] Bray PW, Mahoney JL, Campbell JP (1995) Sensitivity and specificity of ultrasound in the diagnosis of foreign bodies in the hand. J hand Surg Am 20:661–666 10.1016/S0363-5023(05)80287-4 7594298

[9] Dunn IF, Kim DH, Rubin PA (2009). Orbitocranial wooden foreign body: a pre-, intra-, and postoperative chronicle: case report. Neurosurgery.

[10] Jusué-Torres I, Burks SS, Levine CG (2016). Wooden foreign body in the skull base: how did we miss it?. World Neurosurg.

[11] Schlager D, Sanders AB, Wiggins D (1991). Ultrasound for the detection of foreign bodies. Ann Emerg Med.

[12] Kudo S, Takei T (2016). Computed tomography settings for optimal detection of wooden foreign bodies. Am J Emerg Med.

[13] Gilbert FJ, Campbell RS, Bayliss AP (1990). The role of ultrasound in the detection of non-radiopaque foreign bodies. Clin Radiol.

[14] Momose A (2005). Recent advances in x-ray phase imaging. Jpn J Appl Phys.

[15] Pfeiffer F, Weitkamp T, Bunk O (2006). Phase retrieval and differential phase-contrast imaging with low-brilliance x-ray sources. Nat Phys.

[16] Yashiro W, Terui Y, Kawabata K (2010). On the origin of visibility contrast in x-ray Talbot interferometry. Opt Express.

[17] Weitkamp T, Diaz A, David C (2005). x-ray phase imaging with a grating interferometer. Opt Express.

[18] Pfeiffer F, Bech M, Bunk O (2009). x-ray dark-field and phase-contrast imaging using a grating interferometer. J Appl Phys.

[19] Diemoz PC, Endrizzi M, Zapata CE (2013). x-ray phase-contrast imaging with nanoradian angular resolution. Phys Rev Lett.

[20] Lehmann LA, Alvarez RE, Macovski A (1981). Generalized image combinations in dual kVp digital radiography. Med Phys.

[21] Alvarez RE (2011). Estimator for photon counting energy selective x-ray imaging with multibin pulse height analysis. Med Phys.

[22] Ehn S, Sellerer T, Mechlem K (2017). Basis material decomposition in spectral CT using a semi-empirical, polychromatic adaption of the Beer–Lambert model. Phys Med Biol.

[23] Lammers R (1988). Soft tissue foreign bodies. Ann Emerg Med.

[24] Halaas GW (2007). Management of foreign bodies in the skin. Am Fam Physician.

[25] Auweter SD, Herzen J, Willner M (2014). x-ray phase-contrast imaging of the breast - Advances towards clinical implementation. Br J Radiol.

[26] Scherer K, Braig E, Willer K (2015). Non-invasive differentiation of kidney stone types using x-ray dark-field radiography. Sci Rep.

[27] Scherer K, Willer K, Gromann L (2015). Toward clinically compatible phase-contrast mammography. PLoS One.

[28] Velroyen A, Bech M, Tapfer A (2015). Ex vivo perfusion-simulation measurements of microbubbles as a scattering contrast agent for grating-based x-ray dark-field imaging. PLoS One.

[29] Velroyen A, Yaroshenko A, Hahn D (2015). Grating-based x-ray dark-field computed tomography of living mice. EBioMedicine.

[30] Thüring T, Guggenberger R, Alkadhi H (2013). Human hand radiography using x-ray differential phase contrast combined with dark-field imaging. Skeletal Radiol.

[31] Jud C, Braig E, Dierolf M (2017). Trabecular bone anisotropy imaging with a compact laser-undulator synchrotron x-ray source. Sci Rep.

[32] Der BV (2007) Leitlinie der Bundesärztekammer zur Qualitätssicherung in der Röntgendiagnostik. http://www.bundesaerztekammer.de/aerzte/qualitaetssicherung/richtlinien-leitlinien-empfehlungen-stellungnahmen/richtlinien-leitlinien-empfehlungen-zur-qualitaetssicherung/bildgebende-verfahren/roentgendiagnostik/. Accessed 22 Nov 2017

